# Editorial: Emerging strategies for cancer therapy targeting ferroptosis

**DOI:** 10.3389/fonc.2023.1181134

**Published:** 2023-03-20

**Authors:** Manshan Li, Chao Mao, Yanqing Liu, Lianxiang Luo

**Affiliations:** ^1^ The First Clinical College, Guangdong Medical University, Zhanjiang, China; ^2^ Department of Experimental Radiation Oncology, The University of Texas MD Anderson Cancer Center, Houston, TX, United States; ^3^ Herbert Irving Comprehensive Cancer Center, Columbia University, New York, NY, United States; ^4^ The Marine Biomedical Research Institute, Guangdong Medical University, Zhanjiang, China; ^5^ The Marine Biomedical Research Institute of Guangdong Zhanjiang, Zhanjiang, China

**Keywords:** ferroptosis, cancer, lipid peroxidation, drug resistance, biomarkers, targeting strategy

Ferroptosis is an iron-dependent mode of cell death driven by membrane phospholipid peroxidation, which has received increasing attention in cancer research (1). Despite significant advances in cancer treatment, drug resistance in cancer therapy modalities remains a major challenge. How to selectively eliminate cancer cells and overcome resistance to cancer treatments is a main Research Topic. Targeting ferroptosis in cancer cells is an important strategy for cancer treatment and reversing drug resistance (2–4). For example, chimeras synthesized with salomycin derivatives and dihydroartemisinin can induce ferroptosis in resistant pancreatic cancer cells (5). Furthermore, ether phospholipid was found to promote susceptibility to ferroptosis in primary renal and ovarian cancers (6). Ferroptosis is a highly complex and tightly coordinated process characterized by lipid peroxidation and ROS accumulation. Persistently high ROS levels in cancer cells have been found to make cells more sensitive to ferroptosis. Recent studies have reviewed the application of ferroptosis to cancer therapy, tumor resistance, and anti-tumor immunotherapy, demonstrating the potential of inducing ferroptosis to reverse cancer resistance and its promise in the field of cancer therapy (7). Our Research Topic encompasses a comprehensive analysis of 3 review articles and 5 original articles, which delve into the treatment strategy of ferroptosis induced by natural products and potential targets, as well as the research progress of ferroptosis in cancer treatment. This Research Topic effectively showcases the immense potential of ferroptosis in cancer treatment and its ability to reverse cancer drug resistance.

Triggering ferroptosis in cancer cells is a promising strategy for treating cancer and overcoming drug resistance, and at the same time, compounds that induce ferroptosis may open the door to fighting drug-resistant cancers. Recent studies have shown that small molecules can induce cancer ferroptosis through ferroptosis center molecules such as GPX4, Xc- system, and NRF2. These compounds that induce ferroptosis have the common characteristic of high iron activity, which provides ideas for drug development (8, 9). The papers we included suggest that Wogomin, Manolide, and the compound zoledronic acid can induce ferroptosis in cancer cells. Liu et al. confirmed that Wogonin NRF2/GPX4 axis reduces cell GSH levels, thereby enhancing lipid peroxidation and ROS accumulation in pancreatic cancer cells. This strongly confirms the role of ferroptosis in cancer treatment. In addition, Ni et al. found that the natural product Manolide induced ferroptosis in osimertinib-resistant lung cancer cells by inhibiting the NRF2-SLC7A11 axis and mitochondrial calcium ions. More interestingly, Ren et al. showed that zoledronic acid could induce ferroptosis in osteosarcoma cells by increasing HMOX1 protein and mRNA levels, thereby inhibiting the growth of osteosarcoma cells and acting as a cancer therapy. All of these studies strongly suggest that inducing ferroptosis in cancer cells is a promising way to treat cancer and overcome drug resistance.

As ferroptosis show great potential in cancer therapy, it is very necessary to find new biomarkers and therapeutic targets with the good predictive ability of ferroptosis. Recent studies have found that PSTK screened by CRISPR/Cas9 is a key mediator of drug resistance in HCC cells. Reduction in PSTK can lead to GPX4 inactivation, thereby increasing the susceptibility of HCC cells to ferroptosis. Targeting PSTK may be a promising target for inducing ferroptosis in HCC cells and overcoming drug resistance (10). In addition, Huang et al. also found that core genes related to ferroptosis in colon cancer may target cancer cells to produce ferroptosis, which provides a thought basis for the treatment of colon cancer and targeted treatment of cancer resistance. And Xu et al. showed that the prognosis model composed of iron-death related genes could well predict the prognosis of patients with triple-negative breast cancer, and the iron-death genes screened may be a potential new therapeutic target for TNBC, helping to induce ferroptosis of TNBC cells and providing help for cancer treatment.

The development of drug resistance to cancer therapy is a major challenge, and inducing ferroptosis in conjunction with other therapies is currently an effective treatment strategy for preventing acquired drug resistance to cancer therapy. Recent research suggests that resistance to conventional chemotherapy, targeted therapy, and immunotherapy can be overcome by the regulation of ferroptosis (3). Furthermore, Sun et al. ‘s review introduces ferroptosis and its regulatory factors and discusses possible therapeutic approaches for cancer treatment by targeting ferroptosis with nanoparticles. There is growing evidence that the development of nanomaterials targeting ferroptosis is a promising cancer treatment (11). Yun et al. discuss the application and benefits of nanotechnology in combination therapy of tumor immunotherapy and ferroptosis. Their research shows that the use of nanotechnology to deliver drugs targeting ferroptosis can effectively improve the efficacy of ferroptosis in anti-tumor therapy, while at the same time, nanotechnology can integrate various molecules to enhance immune regulation. The field of targeting ferroptosis using nanotechnology and immunotherapy offers exciting directions for the treatment of malignant tumors. Zhang et al. showed that the presence of tumor microenvironments poses considerable obstacles to cancer treatment and that some drugs that target cancer cell metabolism can improve the efficacy of immunotherapy. The addition of nanotechnology can be accurately targeted to minimize systemic side effects caused by immunotherapy. Notably, ferroptosis can stimulate lipid peroxidation in cancer cells and immune cells in the tumor microenvironment, which provides a new prospect for the treatment of ferroptosis in targeted cancers (12, 13). The key to overcoming drug resistance in cancer therapy lies in the development of nanomaterials that exhibit selective cell targeting properties. This strategy is pivotal in facilitating ferroptosis and immunotherapy, both of which play a critical role in the battle against cancer.

The unique metabolism and iron dependence of cancer cells indicate that they are more susceptible to ferroptosis. Inducing ferroptosis is a promising strategy to treat cancer effectively and overcome drug resistance. This topic underscores the importance of ferroptosis-inducing compounds and novel biomarkers in cancer treatment. Moreover, the combination of nanotechnology with ferroptosis and immunotherapy offers new ideas for cancer treatment. However, balancing the susceptibility to ferroptosis of cancer cells, antitumor cells, and immunosuppressive cells in clinical practice poses a significant challenge that needs to be addressed in future research.

## Author contributions

LL conceived and designed the editorial; ML wrote the editorial; LL, YL and CM reviewed the paper and provided comments. All authors read and approved the final manuscript.
